# Clinical Outcomes following Biologically Enhanced Demineralized Bone Matrix Augmentation of Complex Rotator Cuff Repair

**DOI:** 10.3390/jcm11112956

**Published:** 2022-05-24

**Authors:** Ian J. Wellington, Lukas N. Muench, Benjamin C. Hawthorne, Colin L. Uyeki, Christopher L. Antonacci, Mary Beth McCarthy, John P. Connors, Cameron Kia, Augustus D. Mazzocca, Daniel P. Berthold

**Affiliations:** 1 Department of Orthopedic Surgery, University of Connecticut Health Center, Farmington, CT 06032, USA; bhawthorne@uchc.edu (B.C.H.); antonacci@uchc.edu (C.L.A.); mccarthy@uchc.edu (M.B.M.); jconnors@uchc.edu (J.P.C.); ckia@uchc.edu (C.K.); 2 Department of Orthopedic Surgery, Technical University of Munich, 80333 Munich, Germany; lukas.muench@gmail.com (L.N.M.); daniberthold@gmail.com (D.P.B.); 3 Frank H. Netter School of Medicine, Quinnipiac University, Hamden, CT 06518, USA; uyeki@uchc.edu; 4 Massachusetts General Hospital, Massachusetts General Brigham, Harvard Medical School, Boston, MA 02115, USA; amazzocca@mgh.harvard.edu

**Keywords:** shoulder, rotator cuff, allografts, demineralized bone matrix, biologics

## Abstract

Complex rotator cuff tears provide a significant challenge for treating surgeons, given their high failure rate following repair and the associated morbidity. The purpose of this study is to evaluate the clinical outcomes of patients who underwent biologically enhanced demineralized bone matrix augmentation of rotator cuff repairs. Twenty patients with complex rotator cuff tears underwent arthroscopic rotator cuff repair by a single surgeon with demineralized bone matrix (DBM) augmentation that was biologically enhanced with platelet-rich plasma and concentrated bone marrow aspirate. Post-operative MRI was used to determine surgical success. Patient reported outcome measures and range of motion data were collected pre-operatively and at the final post-operative visit for each patient. Ten patients (50%) with DBM augmentation of their arthroscopic rotator cuff repair were deemed non-failures. The failure group had less improvement of visual analogue pain scale (*p* = 0.017), Simple Shoulder Test (*p* = 0.032), Single Assessment Numerical Evaluation (*p* = 0.006) and abduction (*p* = 0.046). There was no difference between the groups for change in American Shoulder and Elbow Society score (*p* = 0.096), Constant-Murley score (*p* = 0.086), forward elevation (*p* = 0.191) or external rotation (*p* = 0.333). The present study found that 50% of patients who underwent biologically enhanced DBM augmentation of their rotator cuff repair demonstrated MRI-determined failure of supraspinatus healing.

## 1. Introduction

Mechanical augmentation using extracellular matrix (ECM) materials—namely in the form of a graft of tissue or synthetic material presents an opportunity for optimizing the healing potential of complex rotator cuff pathologies [1]. These grafts can provide a scaffold for delivering biologic therapies (e.g., platelet-rich plasma (PRP) or concentrated bone marrow aspirate (cBMA)) to augment tendon healing at the operative site while also providing a load-sharing device. This load-sharing and more organized healing environment is thought to prevent scar tissue formation at the tendon-bone interface and encourage the growth of functional tissue comprised of tenocytes, chondrocytes, and osteocytes [1,2].

As a result of the large number of rotator cuff repairs (RCR) performed annually and the high rate of structural failure, considerable efforts have been devoted to developing grafts that augment the RCR site by mechanically reinforcing it as well as providing a biological scaffold that can enhance the rate and quality of the healing process [3]. Because the ECM of the graft directly interacts with tissue microenvironments for stem cell proliferation, it is necessary to consider the design of the patch and how it affects cell differentiation [2]. Prior studies have shown that the composition of microenvironments alters cellular adhesion, differentiation, and morphology [2,4,5,6,7,8]. Since Neviaser et al.’s first use of the interposition allograft for RCR, various graft types have expanded to include synthetic polymers, allograft, autograft, and xenograft materials with varying degrees of clinical success [9]. Common disadvantages to these efforts have included fibrous cartilage formation, strong inflammatory reactions, or rapid degradation of the graft.

Demineralized bone matrix (DBM) is composed of cancellous bone with both osteoinductive and osteoconductive properties. Previous work demonstrated that DBM scaffolding shows excellent adhesion, proliferation, and differentiation of mesenchymal stem cells [10]. Adhesion of these cells to the DBM was maintained even after a simulated arthroscopic mechanical washout stress test. While in vitro testing has shown this material to be an excellent scaffold for biologic augmentation of rotator cuff repairs, few studies have investigated its in vivo efficacy.

Thus, the purpose of the present study was to evaluate the clinical outcomes of patients who underwent biologically enhanced demineralized bone matrix augmentation of rotator cuff repair. It was hypothesized that biologically enhanced demineralized bone matrix augmentation repair would significantly improve shoulder function.

## 2. Materials and Methods

This was a retrospective study. All patients included were older than 18 years of age. Each underwent arthroscopic repair of a complex rotator cuff tear using a DBM scaffold (Flexigraft, Arthrex, Naples, FL, USA) augmented with autogenous PRP and cBMA harvested from the proximal humerus. Surgeries were performed by a single, shoulder fellowship-trained surgeon from September 2015 to December 2017. Institutional review board approval was obtained before the initiation of the study. Patients with RC tear arthropathy (Hamada grade > 3), irreparable massive tears, previous RC surgery requiring tendon transfers, nerve injuries, or pre-operative pseudoparalysis were excluded. Additionally, vulnerable patient populations such as pregnant women and prisoners, as well as individuals with a history of systemic infectious disease (e.g., hepatitis or human immunodeficiency virus) were excluded. All alternative treatment options were discussed with the patient, including continued conservative treatment. Basic demographic information (age, sex, and body mass index) and a thorough medical and surgical history were obtained for each patient.

### 2.1. Imaging

All patients undergoing surgery had a pre-operative magnetic resonance imaging (MRI) of the involved shoulder. On MRI, tendon retraction was quantified on coronal T2 fat-saturated images using the classification system of Patte (A. minimal retraction, B. retraction to humeral head, C. retraction to glenoid) [11]. Fatty infiltration was assessed on T1 sagittal oblique views based on the presence of fatty streaks within the supraspinatus muscle belly using Goutallier’s grading system, which was originally described on computed tomography but is now commonly applied to MRI [12,13].

### 2.2. Surgical Technique

Patients received an interscalene block prior to induction via general anesthesia. Patients were positioned in the beach chair position. First, diagnostic arthroscopy was performed to evaluate the rotator cuff tear and to assess the mobility of the torn edge. For patients that had previously undergone RCR, loose suture material and/or anchors were removed. The graft was prepared by first being soaked in saline at room temperature for at least 30 min prior to use. The 2–3 cc’s of concentrated BMA (cBMA) combined with 2–3 cc’s of PRP were added to the graft.

### 2.3. Bone Marrow Aspirate

Aspiration was performed at the proximal humerus using the Bone Marrow Aspiration Kit (Arthrex) using previously described methods [14]. Four 12-mL double syringes were filled with 2 mL of 1000 U sodium heparin and 9 mL of saline. An 11-gauge non-fenestrated bone marrow aspiration trocar was inserted into the planned site for the first suture anchor at the tendon footprint. The four 12-mL syringes were then used to sequentially aspirate bone marrow from the trocar. Aspirate underwent centrifugation at 800 rpm for 4 min. The upper fractionated layer containing the concentrated bone marrow stromal cells was drawn into the inner syringe. The resulting cBMA from each of the 4 syringes were combined into one syringe.

### 2.4. PRP Concentration

Using the Autologous Conditioned Plasma (ACP) kit (Arthrex, Naples, FL, USA), blood was collected from each patient and then centrifuged at 1500 rpm for 5 min. The concentrated plasma layer was then drawn into a syringe and mixed with the cBMA.

### 2.5. DBM Preparation

The DBM (15 mm × 40 mm × 2 mm) was allowed to soak in saline for a minimum of 30 min prior to use. The cBMA/PRP mixture was then injected into the DBM using a tuberculin syringe. The patch was then soaked for a minimum of 30 min in excess biologic adjuvants.

### 2.6. Repair and Augmentation

After removal of the bone marrow aspiration trocar, the first medial anchor was inserted in its place. Additional anchors were placed as needed. A #2 Fiberwire (Arthrex, Naples, FL, USA) horizontal mattress suture was placed through the DBM graft, the ends of this suture were then passed from the articular side to the bursal side of the torn tendon edge using SutureLasso (Arthrex, Naples, FL, USA). The limbs or the suture were then pulled while the graft was guided into position on the articular side of the torn tendon. Once the DBM was in the proper position, the rotator cuff was repaired in the standard fashion using a double-row technique. Approximately 2 to 5 cc of excess cBMA and PRP were injected into the surrounding tendon. Biceps tenodesis was performed in patients who had pre-operative subpectoral pain. Additionally, subacromial decompression was performed in patients with either a curved or hooked acromion on pre-operative radiographs.

Post-operatively, patients were placed in a 30° abduction sling for 6 weeks. 28 days post-operatively, patients were advanced to 60° active assistive range of motion in external rotation at 30° of abduction and forward elevation from 30° to 180° during physical therapy. Patients were allowed to initiate an active assistive range of motion in external rotation and forward elevation without limitations until 12 weeks post-operatively. At 12 weeks, patients began isometric strengthening of the rotator cuff muscles with progression to isotonic strengthening at 18 weeks.

### 2.7. Clinical Outcome Measures

Simple Shoulder Test (SST), American Shoulder and Elbow Surgeons (ASES), Single Assessment Numerical Evaluation (SANE), visual analog pain scale (VAS), and Constant-Murley (CM) scores were collected pre-operatively and at the final post-operative visit for each patient. The change in these scores was calculated for each patient.

### 2.8. Determination of Surgical Outcome

Patients were divided into either surgical success or surgical failure groups for data analysis. To accomplish this, a one-year post-operative MRI was used to determine if the supraspinatus tendon successfully healed. For some patients, an earlier post-operative MRI was ordered if there was a concern of surgical failure. Five patients did not have MRIs post-operatively due to their high degree of clinical improvement. These five were considered surgical successes.

### 2.9. Statistical Methods

Descriptive statistics were calculated as a mean and standard deviation or frequency and proportion for each group. Independent values student’s t-tests were used to compare numerical data, and chi-square analysis with Fischer’s exact tests was used to compare categorical data. 95% confidence intervals (CIs) were calculated. Missing data were excluded from the analysis. A *p*-value of less than 0.05 was considered to be statistically significant. All studies were performed using SPSS (version 28, IBM, Armonk, NY, USA) statistical software.

## 3. Results

**Figure 1:**
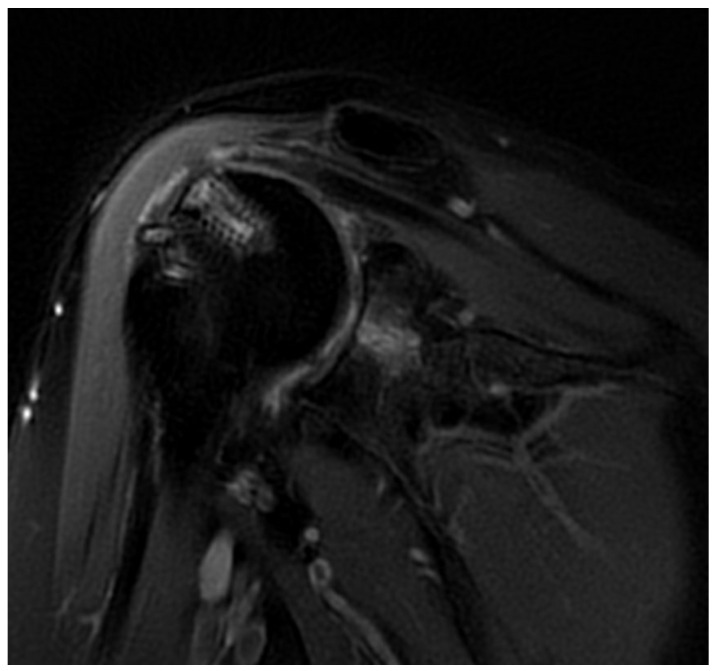
T2-weighted sagittal MRI of a shoulder following successful healing of a supraspinatus tear with DBM, PRP, and cBMA augmentation.

Twenty total patients underwent RCR with DBM. Of the 20, 10 patients demonstrated failure of their repair on post-operative MRI, 5 patients demonstrated an intact repair on the post-operative MRI and 5 did not receive a postoperative MRI given their excellent clinical improvements (Figure 1). The five subjects that did not have a post-operative MRI were considered non-failures.

There were no differences between the success and failure groups for age, gender, body mass index (BMI), or diabetes status (Table 1). There were no patients with rheumatologic conditions or a history of cancer. There were no statistically significant differences between groups on handedness, surgical side, Patte Classification (tendon retraction), Goutallier Stage (fatty infiltration), history of prior shoulder surgery of any type, or history of prior rotator cuff repair (Table 1). Of the 10 non-failure patients, 1 had an acute tear while 9 were chronic. All the failed patients had chronic tears. Biceps tenodesis was performed concomitantly with the DBM repair in 10% of the non-failure group and 80% of the failure group, which was significantly different. Subacromial decompression was performed in 20% of non-failure patients and 30% of failure patients, which was not significantly different (Table 1).

There was no difference between the failure and non-failure groups for pre-operative VAS, ASES, SST, SANE, or CM scores. Additionally, there was no difference between groups for pre-operative forward elevation, abduction, or external rotation (Table 2).

The non-failure group had a greater post-operative decrease in pain (*p* = 0.017; CI: −5.4 to −6.1) compared to the failure group. The failure group also showed significantly worse post-operative improvements in SST (*p* = 0.032; CI: 0.2 to 5) and SANE (*p* = 0.006; CI: 15.8 to 79.6) (Table 3). There was no difference between the two groups for change in ASES (*p* = 0.096; CI: −3.7 to 41.6) and CM score (*p* = 0.086; CI: −3.5 to 46.3) though these approached significance. The non-failure group had a significantly greater improvement in abduction (*p* = 0.046; CI: 1 to 84), but there was no difference in forward elevation (*p* = 0.191; CI: −15 to 69) or external rotation (*p* = 0.333; CI: −9 to 26) (Table 3). There was no difference in follow up between the non-failure group (13.1 ± 6.3 months) and the failure group (13.5 ± 6.9 months) (*p* = 0.894; CI −6.6, 5.8).

## 4. Discussion

In the present study, patients treated with DBM augmented with cBMA and PRP for complex rotator cuff tears had a failure rate of 50%. There were no pre-operative differences in comorbidities or patient-reported outcome measures (PROM) between those with clinical rotator cuff repair failure and those who did not fail. However, there was a difference in rates of concomitant biceps tenodesis, with those in the failure group having undergone more of this procedure. The patients who did suffer failure expectedly had less improvement of PROMs than those who did not fail. All patients that failed repair ultimately required further revision surgery or went on to reverse shoulder arthroplasty.

Failure after rotator cuff repair is a common problem that complicates the treatment of this pathology. This is particularly true for chronic tears, revision surgeries, and complex-massive tears for which failure of repair is between 39.8 and 70% [15,16,17,18,19,20]. Biologic augmentation of these complex cases presents a possible option for decreasing the risk of this poor outcome [21,22,23]. Thon et al. found high rates of healing with the use of a bio-inductive collagen patch scaffold during the repair of massive rotator cuff tears [23]. Recent studies have also found lower failure rates in small and medium-sized tears augmented with PRP during the repair [24,25]. Additionally, cBMA has been found to significantly decrease rotator cuff repair failure rates [26,27,28].

In animal models, DBM augmentation for bone-tendon healing has shown promising results. Sundar et al. demonstrated the DBM augmented patellar tendon repair in an ovine model showed fewer failures when compared to non-augmented repairs at 12 weeks [29]. Mouse and rabbit models for rotator cuff repair have shown similar efficacy in DBM augmentation [30,31]. Smith et al. demonstrated that DBM augmented with PRP showed improved tendon-to-bone healing in large, retracted rotator cuff tears at 12 weeks [32]. The failure rate observed in our study is similar to the rates previously described for complex rotator cuff repairs, it is unclear to what degree this was impacted by the use of DBM [15,16,17,18,19,20]. This may draw concern that DBM and similar constructs may not significantly improve the healing of rotator cuff tears in humans.

While complex rotator cuff repair augmentation with DBM may have decreased the failure rate for this procedure, it is difficult to draw definitive conclusions regarding the impact of DBM on these repairs. This study was limited by its limited sample size and a lack of a comparison group who underwent standard repair without DBM augmentation. It is impossible to determine how this augmentation system impacts healing rates without utilizing a randomized-control methodology. Furthermore, there may have been selection bias in choosing patients who would be treated with DBM augmentation. Another limitation is the concomitant use of cBMA and PRP in these repairs. These additional augments were used as this is the current practice of the treating surgeon. As such, this case series addresses the success rate for DBM augmented with PRP and cBMA rather than the success rate of DBM alone. Furthermore, post-operative ASES scores were not available for three patients (one failure, two non-failure) and CM scores were not available for six patients (one failure, five non-failure). However, the pre-operative patient-reported measures, rather than the post-operative measures, are more meaningful for this study to ensure that there were no pre-operative differences between the failure and the non-failure groups. Finally, post-operative MRIs were not available for every patient, with five patients missing these. These five patients all showed significant clinical improvement post-operatively, and as such, an MRI was not obtained. These patients were deemed successes for the purpose of this study, though it is possible that some of these patients had asymptomatic retears. Ultimately, as full determination of the efficacy of biologically enhanced DBM as an augment for rotator cuff repairs is difficult with a retrospective case series, a prospective study, ideally, a randomized control trial, comparing those treated with this form of augmentation compared to those treated without would be ideal.

## 5. Conclusions

The present study found that 50% of patients who underwent biologically enhanced DBM augmentation of their rotator cuff repair demonstrated MRI-determined failure of supraspinatus healing. While this failure rate is similar to rates previously reported for similar tears it is difficult to conclude how much of an impact DBM augmentation had on overall healing. Further investigation, ideally with a randomized control study, is needed to determine the true impact of biologically enhanced DBM for the augmentation of rotator cuff repairs.

## Figures and Tables

**Table 1:** Demographic and Injury Information for Non-Failure and Failure Patients.

		Non-Failure (*n* = 10)	Failure (*n* = 10)	*p*-Value	95% CI	
					Lower	Upper
Age (years ± SD)		58.6 ± 4.9	51.3 ± 10.2	0.056	−0.2	14.8
Gender (% Female)		40	40	1		
BMI (kg/m^2^ ± SD)		27.6 ± 3.6	28.1 ± 3.7	0.754	−3.9	2.9
Smoking (%)		10	30	0.582		
Diabetes Mellitus (%)		30	10	0.582		
Rheumatologic Condition		0	0			
Cancer		0	0			
Handedness (% RHD)		90	100	0.305		
Surgical Side (% Right side)		60	70	0.639		
Chronic Tear (% Chronic)		90	100	0.305		
Primary Repair (% Primary)		40	30	0.639		
Patte Classification	A	5	4	0.637		
	B	3	5			
	C	0	0			
	NC	2	1			
Goutallier Classification	0	0	0	0.134		
	1	5	2			
	2	3	4			
	3	0	2			
	4	0	1			
	NC	2	1			
Previous Shoulder Surgery	0	4	2	0.281		
	1	6	4			
	2	0	2			
	3	0	1			
	4	0	1			
Previous RCR	0	4	3	0.315		
	1	6	4			
	2	0	1			
	3	0	2			
Biceps Tenodesis (%)		10	80	0.005 *		
SAD (%)		20	30	0.606		

BMI = body mass index; NC = not classified; RCR = rotator cuff repair, SAD = subacromial decompression; CT = Confidence Interval; * = *p* < 0.05.

**Table 2:** Pre-operative Pain and Functional Measurements.

	Non-Failure (*n* = 10)	Failure (*n* = 10)	*p*-Value	95% CI	
				Lower	Upper
VAS	5.8 ± 2.5	6.9 ± 2.1	0.302	−3.3	1.1
ASES	30.5 ± 19.3	29.6 ± 14.4	0.17	−5.1	26.9
SST	4.2 ± 3.3	2.5 ± 2.1	0.187	−0.9	4.3
SANE	8.3 ± 5.9	8.0 ± 7.4	0.921	−6	6.6
CM	43.0 ± 19.1	35.8 ± 8.4	0.289	−6.6	21
Forward Elevation	133 ± 44	119 ± 26	0.403	−20	48
Abduction	123 ± 46	100 ± 29	0.336	−19	53
External Rotation	42 ± 21	35 ± 8	0.341	−8	22

VAS = visual analogue scale; ASES = American Shoulder and Elbow Surgeons Shoulder Score; SST = Simple Shoulder Test; SANE = Single Assessment Numeric Evaluation; CM = Constant-Murley; CI = Confidence Interval.

**Table 3:** Post-operative Change in Pain and Function Scores.

	Non-Failure (*n* = 10)	Failure (*n* = 10)	*p*-Value	95% CI	
				Lower	Upper
VAS (*n* = 10,10)	−3.6 ± 3.1	−0.06 ± 1.9	0.017 *	−5.4	−0.6
ASES (*n*= 8,9)	29.6 ± 23.4	10.8 ± 20.3	0.096	−3.7	41.4
SST (*n*= 10,10)	3.7 ± 3.1	1.1 ± 1.8	0.032 *	0.2	5
SANE (*n* = 10,10)	68.0 ± 28.9	20.3 ± 38.5	0.006 *	15.8	79.6
CM (*n*= 5,9)	16.4 ± 16.3	−5.0 ± 22.3	0.086	−3.5	46.3
Forward Elevation (*n* = 10,10)	22 ± 29	−5 ± 56	0.191	−15	69
Abduction (*n* = 10,10)	26 ± 26	−16 ± 57	0.046 *	1	84
External Rotation (*n* = 10,10)	12 ± 13	3 ± 24	0.333	−9	26

VAS = visual analogue scale; ASES = American Shoulder and Elbow Surgeons Shoulder Score; SST = Simple Shoulder Test; SANE = Single Assessment Numeric Evaluation; CM = Constant-Murley; CI = Confidence Interval; * = *p* < 0.05.

## Data Availability

Not applicable.
